# Impact of Eutectic Solvents Utilization in the Microwave Assisted Extraction of Proanthocyanidins from Grape Pomace

**DOI:** 10.3390/molecules27010246

**Published:** 2021-12-31

**Authors:** Rodrigo T. Neto, Sónia A. O. Santos, Joana Oliveira, Armando J. D. Silvestre

**Affiliations:** 1 CICECO—Aveiro Institute of Materials, Department of Chemistry, Campus Universitário de Santiago, University of Aveiro, 3810-193 Aveiro, Portugal; rtneto@ua.pt (R.T.N.); santos.sonia@ua.pt (S.A.O.S.); 2 REQUIMTE—Laboratório Associado para a Química Verde, Department of Chemistry and Biochemistry, Faculty of Sciences, University of Porto, Rua do Campo Alegre, 687, 4169-007 Porto, Portugal; jsoliveira@fc.up.pt

**Keywords:** proanthocyanidin, eutectic solvents, microwave assisted extraction, by-products, grape pomace

## Abstract

The extraction of proanthocyanidins (PACs), despite being an important and limiting aspect of their industrial application, is still largely unexplored. Herein, the possibility of combining eutectic solvents (ESs) with microwave assisted extraction (MAE) in the extraction of PACs from grape pomace (GP) is explored, aiming to improve not only the extraction yield but also the mean degree of polymerization (mDP). The combination of choline chloride with lactic acid was shown to be the most effective combination for PACs extraction yield (135 mg_PAC_/g_GP_) and, despite the occurrence of some depolymerization, also enabled us to achieve the highest mDP (7.13). Additionally, the combination with MAE enabled the process to be completed in 3.56 min, resulting in a considerably reduced extraction time.

## 1. Introduction

Proanthocyanidins (PACs), or condensed tannins, are polymeric phenolic compounds that consist of flavan-3-ol monomers and its derivatives, and can be found throughout the plant kingdom [1]. Their function in plants is believed to be mostly related to defense against microbial pathogens [2] and herbivores [3]. Beyond that, PACs have several industrial applications such as tanning agents in leather production, as adhesives in wood agglomerates, and as additives in wine maturation [4].

The PAC obtention process for industrial applications is based on their extraction from dedicated crops such as quebracho heartwood, with a hot aqueous sulfite solution [5]. Despite being widely used, this method has a low extraction yield (Y_PAC_) and results in extracts with a low mean degree of polymerization (mDP) [6], rendering PAC extracts very expensive, which limits their use in industrial applications.

Considering the role that PACs might have in the replacement of hazardous chemicals in the production of leather or wood agglomerates, the development of new, sustainable, and more efficient extraction methodologies is of the utmost importance. To address the current limitations, sustainable PAC sources need to be found and more efficient extraction methodologies must be developed.

As far as raw materials are concerned, there is increasing evidence of the potential of agroforestry by-products as reliable, low-cost alternative sources for PACs due to their high PAC content, availability, and possibility of being locally and sustainably sourced [7]. In addition, the use of by-products from other agroforestry activities fits into the circular economy concept in which by-products are reused and extracted as much as possible before being discarded as waste [8]. From the available options, grape pomace (GP), a by-product from the wine industry, comes out as one of the most interesting sources of PACs, since in 2018 alone, 292 million hL of wine were produced [9], corresponding to 11 million tons of grape pomace that have no added value application, despite presenting high PAC content [10].

The PAC extraction process, particularly from GP, can be improved by exploring two routes, namely, neoteric solvents such as eutectic solvents (ESs) and auxiliary extractive techniques such as microwave assisted extraction (MAE).

ESs used as a biomass processing solvent have increased in popularity due to their low price and toxicity, ease of preparation, and possibility of being tailor-made for a specific purpose [11]. ESs are generally described as binary systems composed of a hydrogen bond acceptor (HBA) and hydrogen bond donor (HBD) that have an eutectic point (composition at which the melting temperature is the lowest) and differ from deep eutectic solvents (DES) in following the behavior of an ideal mixture [12].

PAC extraction with ESs was previously explored for different raw materials such as *Ginkgo biloba* leaves from which it was possible to extract 22.1 mg_PAC_/g using a mixture of choline chloride and malonic acid at a molar ratio of 1:2 and with a water content of 55% (*w*/*w*) [13], and white grape pomace from which 125.9 mg_PAC_/g was obtained using a mixture with mass fractions of 0.5, 0.3, and 0.2 of choline chloride, water, and ethanol, respectively [14]. In addition, it was also shown that by varying the composition of a quaternary eutectic system (choline chloride, glycerol, water, and ethanol), the characteristics of the final grape pomace extract such as mDP and galloylation percentage (%Gal) can be controlled with values ranging from 5.9 and 30.6%, respectively, with the ES composition mentioned previously from 7.5 and 47.2%, respectively, using an ES mixture of glycerol, water, and ethanol with mass fractions of 0.68, 0.05, and 0.27, respectively [14].

MAE has been described as a good candidate in the extraction of PACs from agroforestry by-products in combination with conventional solvents such as ethanol:water mixtures in the extraction of PACs from maritime pine bark [15]. Additionally, neoteric solvents such as ionic liquids have also been suggested in the extraction of PACs from cortex cinnamomic with a 1.25 M aqueous solution of 3-methylimidazolium bromide that resulted in an yield improvement of 125% when compared to the conventional MAE combined with water [16]. To the best of our knowledge, the use of ESs in the extraction of PACs with MAE has not yet been explored.

Herein, we explore the stability and extractability of PACs in ESs as well as the stability of ESs when subjected to MAE. After selecting the combination of choline chloride with lactic acid or glycerol as the best candidates in MAE of PACs, the impact that the content of each ES component has on relevant parameters of the final extract such as Y_PAC_, mDP, and %Gal was assessed. Additionally, the carbohydrate yield (Y_CH_) obtained for each condition was also determined in order to understand the possible contaminations with other macroconstituents. Similarly, the impact that MAE conditions, namely temperature (T), biomass percentage (%BM), and extraction time (t) have on the mentioned parameters of the final extract was also assessed.

## 2. Results and Discussion

### 2.1. Screening of Proanthocyanidin Stability in Eutectic Solvents

PACs have been described to undergo depolymerization under heat and acidic conditions [17] and to degrade under mild alkaline conditions [18]. Therefore, it is important to assess the impact that each ES has on PACs under normal operating MAE conditions. In order to determine this, solutions of 20 mg_PAC_/g_ES_ of PAC reference material obtained from white GP were prepared in ESs composed of 37.5% (*m*/*m*) of HBA (choline chloride—ChCl; betaine—Bet; proline—Pro), HBD (urea—Ur; malic acid—MalA; lactic acid—LacA; citric acid—CitA; glucose—Glu; glycerol—Glyc), and 25% (*m*/*m*) of water.

**Figure 1:**
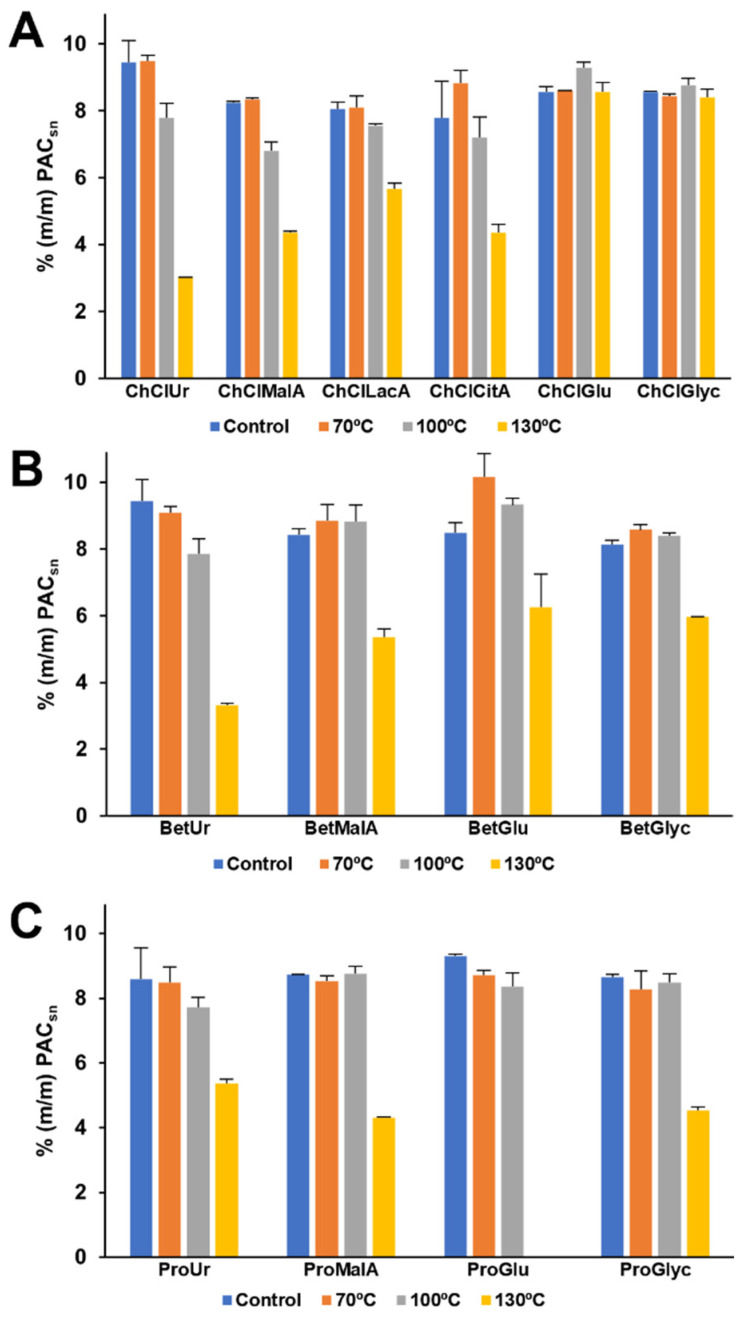
Quantification of PACs solubilized in different ESs and incubated in MAE at different temperatures. (**A**) ChCl-based; (**B**) Bet-based; (**C**) Pro-based.

The reference material was then incubated at 70, 100, or 130 °C for 10 min in the selected ESs, and the PAC content was determined by the acidic butanol assay with the results being presented in Figure 1.

As can be seen in Figure 1, when Ur is used as HBD, PAC degradation can be observed above 100 °C, especially when paired with ChCl, with this effect being even more evident at 130 °C where the total PAC content is reduced to one third of the initial amount for ChCl:Ur and Bet:Ur while Pro:Ur experiences only a decrease of 37%. This is in line with what would be expected considering the alkaline character of these Ur-based ESs [18].

When using MalA as HBD, no degradation was detected up to 100 °C except when combined with ChCl (Figure 1A) and all HBAs led to the degradation of half of the PAC content at 130 °C, which is in agreement with what was previously described for strong acid media [17]. Comparing the combination of ChCl with other organic acids such as LacA and CitA (Figure 1A), it can be observed that despite presenting similar degradation values at 100 °C, LacA leads to significantly less degradation at 130 °C when compared to the other tested organic acids. This might be related to the fact that LacA is a monocarboxylic acid while MalA and CitA are dicarboxylic and tricarboxylic acids, respectively, which in turn leads to an increasing molar concentration of carboxylic groups per unit mass of ES, 4.16 × 10^−3^, 5.60 × 10^−3^, and 5.85 × 10^−3^ mol/g_ES_, respectively.

A wide range of outcomes can be observed when using Glu as HBD, particularly that no degradation was observed when paired with ChCl regardless of temperature (Figure 1A), mild degradation at 130 °C when paired with Bet (Figure 1B), and complete degradation at 130 °C when paired with Pro (Figure 1C). This is probably mostly related with degradation induced by the by-products resulting from the Maillard reactions that are expected to occur in ES containing amino acids and sugars when exposed to heat [19]. Similarly, when using Glyc as HBD, no degradation was observed up to a temperature of 100 °C for all HBAs and with ChCl at 130 °C (Figure 1A), mild degradation at 130 °C with Bet (Figure 1B), and PAC reduction to half of the initial amount with Pro (Figure 1C).

In summary, it can be concluded that in general, the use of ChCl-based ESs is less prone to PAC degradation, from which the combination with Glu or Glyc have the best results (no degradation). Additionally, the use of LacA was shown to be less prone to degradation at high temperatures when compared with the remaining organic acids. 

### 2.2. Screening of Eutectic Solvents

**Figure 2:**
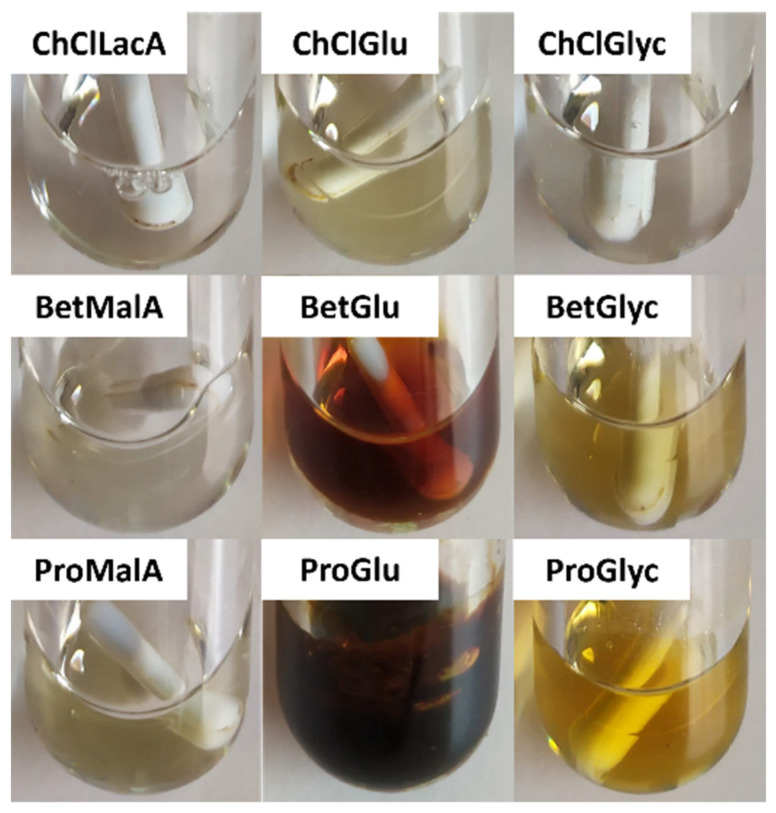
ESs after incubation in MAE at 130 °C for 10 min.

In addition to assessing the PAC stability in the tested ESs, it is also important to determine the stability of the ESs themselves during the MAE process. Therefore, all the ESs were incubated for 10 min at 130 °C and the relevant results are presented in Figure 2.

As can be observed, the combinations of all HBAs with organic acids were stable as far as visual analysis is concerned. Nevertheless, side reactions between ChCl and organic acids have been previously reported, especially for MalA, with which it is possible to achieve 17% (mol%) of ChCl esterification after 2 h at 100 °C and to a lesser extent with LacA, which achieved 7% in the same conditions [20]. When using Glu as HBD, some color development is always present, especially when combined with Bet and Pro, most likely due to the formation of melanoidins during Maillard reactions [19]. Similar observations were made with Glyc, although not as prevalent and were considered nonexistent when paired with ChCl.

To sum up, the use of ChCl-based ESs appears to give rise to more stable solvents when compared to Bet and Pro, and HBDs such as Glu seem to be very thermally sensitive and therefore should be avoided in situations in which temperature in necessary.

### 2.3. Screening of Proanthocyanidin Extractability with Eutectic Solvents

PAC extractability, or the ability to remove the compound of interest from the matrix in which it is present and effectively keep it in solution, is of the utmost importance in the development of an extraction process. To screen the effectiveness of each candidate for this purpose, 10% (*m*/*m*) of GP was suspended in the selected ESs and subjected to MAE for 10 min at 100 °C, followed by centrifugation and recovery of the supernatant.

**Figure 3:**
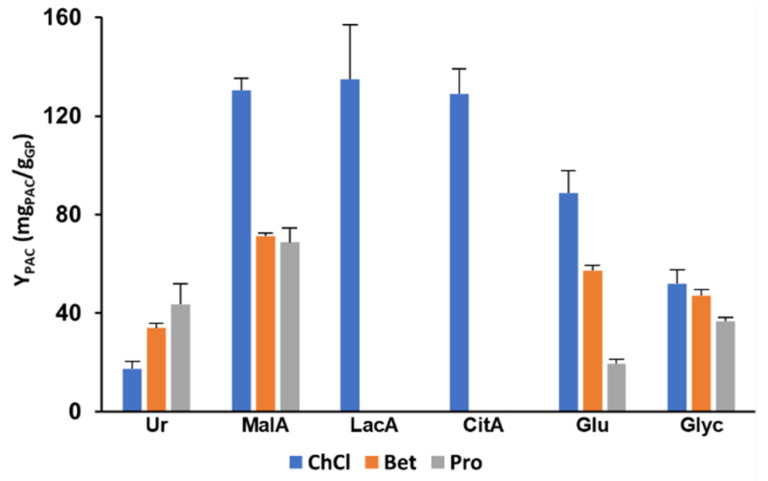
Extractability of PACs from GP with different ESs using MAE at 100 °C for 10 min.

In Figure 3, it can be observed that, in general, the screened ESs followed the same trend, with the ones containing ChCl as HBA leading to higher extraction yields than the remaining ones, which is in general agreement with the findings of Cao et al. [13] that compared several ChCl and Bet-based ESs. Nevertheless, when Ur is used as HBD, the best results were obtained with Pro as HBA (43.7 mg_PAC_/g_GP_), followed by Bet and ChCl.

The best extractability results were obtained by combining MalA with ChCl (130.5 mg_PAC_/g_GP_), doubling from what was obtained with Bet or Pro. When analyzing the effect of combining different organic acids with ChCl, namely, LacA and CitA, it becomes clear that for this system, no significant differences were observed with very similar values being obtained of 135.0 and 129.1 mg_PAC_/g_GP_, respectively. The use of ESs based on ChCl and organic acids, although not widely explored for the extraction of PACs, is frequently found to be the most effective combination when compared to others not containing organic acids for the extraction of phenolic compounds from biomass such as chlorogenic acid from *Morus alba* L. leaves using ChCl:CitA [21], anthocyanins, and catechin from grape skin using ChCl with oxalic acid [22] and pelargonidin-3-glucoside from strawberry extrudate using ChCl with glycolic and oxalic acids [23]. In the case of PACs, this may be explained by two factors, namely due to PAC depolymerization that reduces their affinity for the pectin and hemicellulose fractions of the cell wall [24] or by dissolving the hemicellulose or lignin components of the cell wall contributing to the PAC release into solution [25].

Similarly, when using Glu or Glyc, the best results were also obtained by combining them with ChCl, resulting in extraction yields of 88.8 and 51.8 mg_PAC_/g_GP_, respectively. The use of polyols in ESs such as Glu or Glyc has been extensively explored and are considered good solvents for phenolic compounds due to the high number of hydroxyl groups [26]. Nevertheless, the extraction yields obtained here are considerably lower than those obtained with carboxylic acids. Interestingly, the use of Glu is considerably more affected by the combination of different HBAs than Glyc, which, as shown previously, could be related with its degradation when combined with Bet or Pro.

Based on the results obtained previously, ChCl:LacA and ChCl:Glyc were chosen as the best candidates for solvent composition optimization. This is justified by the thermal stability exhibited by both ESs, by the high extractability of ChCl:LacA, and comparatively low PAC degradation at high temperatures when compared with the other organic acids, and by the inexistence of PAC degradation at all tested temperatures with ChCl:Glyc.

### 2.4. Solvent Composition Optimization

Although ChCl:LacA and ChCl:Glyc presented good extraction characteristics, it should be noted, as described in our previous work [14], that the variation of each ES component’s content has a great impact on the characteristics of the final extract, affecting not only extraction yield but also the mDP and galloylation percentage (%Gal).

**Figure 4:**
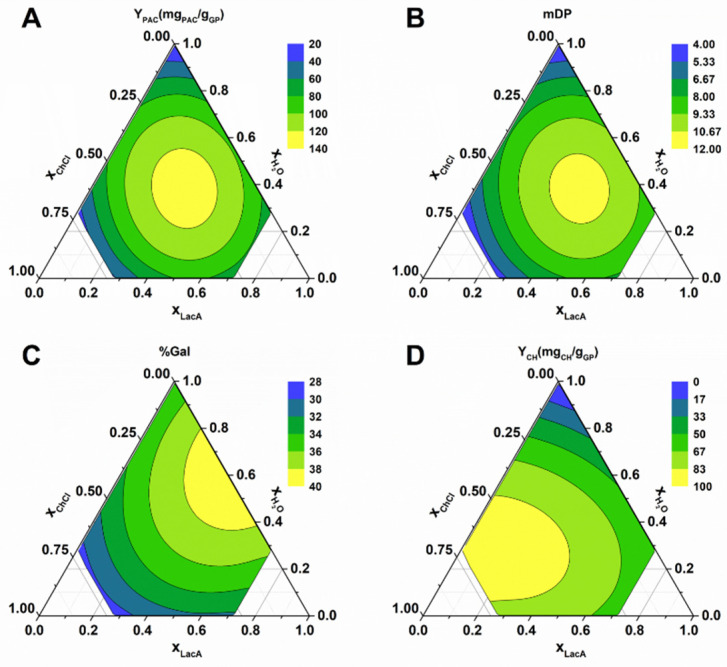
Contour plots obtained for solvent composition optimization of ChCl:LacA. (**A**) Y_PAC_; (**B**) mDP; (**C**) %Gal; (**D**) Y_CH_.

In order to determine the effect of each component on the variables mentioned previously, response surface methodology (RSM) was employed with 16 experimental points in which the mass fractions were varied from 0 to 0.7 for ChCl, 0 to 0.9 for LacA, and 0 to 0.7 for water, as specified in Table S2 of Supplementary Materials. For each experimental condition, Y_PAC_, mDP, %Gal, and Y_CH_ were determined. The corresponding polynomials are depicted in Equations (1)–(4), and the respective contour plots shown in Figure 4.

Y_PAC_ = −31.83x_ChCl_ + 17.69x_LacA_ − 102.3x_water_ + 414.6x_ChCl_x_LacA_ + 589x_ChCl_x_water_ + 486.1x_LacA_x_water_ (1)

mDP = 2.402x_ChCl_ + 3.875x_LacA_ - 4.181x_water_ + 26.45x_ChCl_x_LacA_ + 34.81x_ChCl_xwater + 29.75x_LacA_x_water_ (2)

%Gal = 27.67x_ChCl_ + 34.20x_LacA_ + 22.18x_water_ + 32.57x_ChCl_x_LacA_ + 26.14x_ChCl_x_water_ + 17.50x_LacA_x_water_ (3)

YC_H_ = 20.63x_ChCl_ − 3.584x_LacA_ + 64.2x_water_ + 178.3x_ChCl_x_LacA_ + 124.5x_ChCl_x_water_ + 218.9x_LacA_x_water_ (4)

The performed ANOVA analysis revealed that the resulting models for Y_PAC_, mDP, %Gal, and Y_CH_ all had *p*-values < 0.002, adjusted r^2^ values of 0.80, 0.82, 0.82, and 0.75, respectively, and predicted r^2^ values of 0.70, 0,73, 0.67, and 0.57, respectively, indicating the statistical robustness of the resulting quadratic models.

As observed in Figure 4A,B, the optimal solvent composition for Y_PAC_ and mDP was very similar (Y_PAC_ − x_ChCl_ = 0.34, x_LacA_ = 0.39, x_water_ = 0.27; mDP − x_ChCl_ = 0.38, x_LacA_ = 0.39, x_water_ = 0.23). This result is important because it demonstrates that the two variables can be maximized under the same conditions. The result is also interesting considering that under these conditions, the mixture develops a slight red color normally associated with PAC depolymerization [17], which could be indicative of a decrease in the final extract’s mDP. Nevertheless, the reduction in mDP was not observed, which might be related to the high acidic character of the solvent that leads to the depolymerization of PACs with very high mDP (20 or higher, especially the ones present in grape skin [27]), resulting in the release of PACs with mDP around 10 into solution that as mentioned previously, can be explained by the reduction in PAC’s affinity for the pectin and hemicellulose fractions of the cell wall [24]. Another possible explanation previously mentioned is the solubilization of the hemicellulose or lignin components of the cell wall contributing to the PAC release into solution [25], which would contribute in a similar way to the release of high mDP PACs.

By maximizing the resulting models for Y_PAC_ and mDP, the best solvent composition is x_ChCl_ = 0.36, x_LacA_ =0.39 and x_water_ = 0.25, and the expected results were 128.5 mg_PAC_/g_GP_ and a mDP of 11.1. Nevertheless, the results of the confirmation runs were 152.4 mg_PAC_/g_GP_ and a mDP of 8.4, which might be indicative that when using this solvent system, additional PAC depolymerization should be expected to occur if extraction conditions are not carefully controlled.

The effect of the ES composition in other characteristics such as %Gal and Y_CH_ can also be observed in Figure 4C,D, from which one can conclude that the highest %Gal values were obtained without water addition to the ES and that the opposite was observed for Y_CH_, respectively. This could be explained by the fact that when water is added to the ES dissociation and the ionization of ChCl and LacA, respectively, occurs, this leads to an increase in solvent polarity that contributes to the solubilization of non-galloylated PACs and carbohydrates. Interestingly, the maximum YPAC obtained (Figure 4A) seems to be a combination of the high carbohydrate solubilization was obtained with high x_water_ and a x_LacA_ between 0.2 and 0.4 (Figure 4D), and high %Gal was obtained with low x_water_ and x_ChCl_ between 0.3 and 0.5 (Figure 4C).

**Figure 5:**
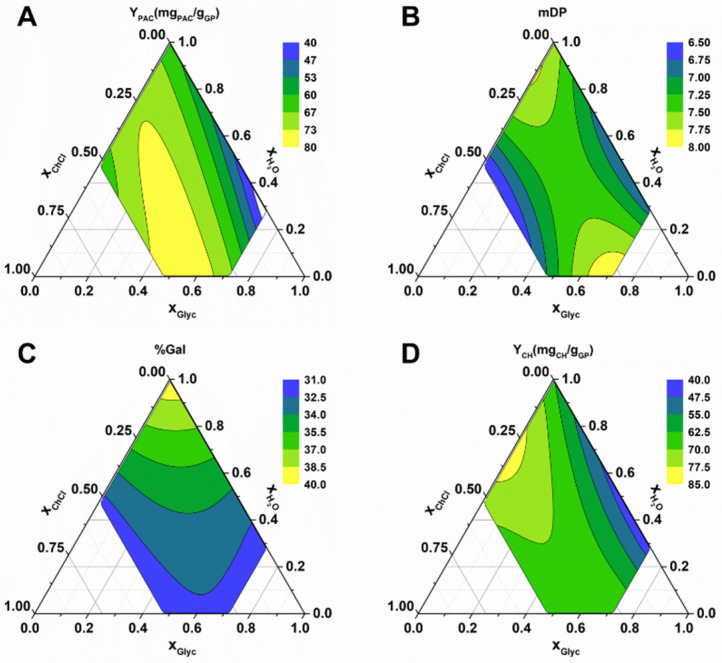
Contour plots obtained for solvent composition optimization of ChCl:Glyc. (**A**) Y_PAC_; (**B**) mDP; (**C**) %Gal; (**D**) Y_CH_.

Similar experiments were conducted for ChCl:Glyc in which the mass fractions were varied from 0 to 0.7 for ChCl, 0 to 1 for Glyc, and 0 to 0.5 for water, as specified in Table S3 in the Supplementary Materials. For each experimental condition, Y_PAC_, mDP, %Gal, and Y_CH_ were determined. The corresponding polynomials are depicted in Equations (5)– (8), and the respective contour plots are shown in Figure 5.

Y_PAC_ = 15.65x_ChCl_ + 62.0x_Glyc_ − 20.60x_water_ + 28.21x_ChCl_x_Glyc_ + 313.7x_ChCl_x_water_ + 166.7x_Glyc_x_water_ (5)

%Gal = 7.53x_ChCl_ + 7.65x_Glyc_ + 1.205x_water_ − 3.177x_ChCl_x_Glyc_ + 11.08x_ChCl_x_water_ + 8.60x_ChCl_x_water_ (6)

mDP = 29.06x_ChCl_ + 39.48x_Glyc_ + 23.22x_water_ − 2.805x_ChCl_x_Glyc_ + 23.32x_ChCl_x_water_ + 3.451x_ChCl_x_water_ (7)

Y_CH_ = 49.18x_ChCl_ + 63.2x_Glyc_ − 1.290x_water_ − 46.41x_ChCl_x_Glyc_ + 168.6x_ChCl_x_water_ + 171.8x_ChCl_x_water_ (8)

ANOVA analysis showed that the resulting models for Y_PAC_, mDP, %Gal, and Y_CH_ all had *p*-values < 0.002, adjusted r^2^ values of 0.79, 0.95, 0.86, and 0.73, respectively, and predicted r^2^ values of 0.61, 0,90, 0.77, and 0.53, respectively, which indicates the statistical robustness of the resulting quadratic models.

As can be observed in Figure 5A,B, Y_PAC_ and mDP were both negatively affected by increasing Glyc mass fraction, with the optimal solvent compositions not including it (YPAC − x_ChCl_ = 0.56, x_Glyc_ = 0.0, x_water_ = 0.44; mDP − x_ChCl_ = 0.70, x_Glyc_ = 0.0, x_water_ = 0.30). Interestingly, and contrary to what happens with ChCl:LacA, the observed behavior for Y_PAC_ had a maximum that followed a ridge with roughly constant water content and with varying mass fraction values of ChCl and Glyc (Figure 5A). This was in agreement with our previous findings [14] and allows for the obtention of PAC extracts with different values of mDP and %Gal with minimal loss of Y_PAC_. Contrary to what was observed for ChCl:LacA, no PAC depolymerization was visually observed (based on the absence of red color). Despite that fact, the resulting Y_PAC_ and mDP values were considerably lower, further sustaining the importance of PAC depolymerization/lignin and hemicellulose solubilization in their extraction process.

If the results are maximized for Y_PAC_ and mDP, the ideal solvent composition is x_ChCl_ = 0.65, x_Glyc_ = 0.0, and x_water_ = 0.35 and the expected results are 74.0 mg_PAC_/g_GP_ and a mDP of 7.85. Interestingly, the confirmation runs returned values that were much closer to what was expected from the models than what was observed for LacA, further sustaining the advantage of using ESs that do not contribute to PAC depolymerization.

As far as %Gal and Y_CH_ are concerned (Figure 5C,D), water content appeared to have a similar effect to what was observed with ChCl:LacA with small differences in %Gal that can be attributed to Glyc. More specifically, Glyc, having several hydroxyl groups, has a great effect on the extraction of PACs with galloylated subunits, as shown by our previous work [14] in which it was demonstrated that the increase in ethanol content is directly proportional to the %Gal.

One aspect that is also relevant in the development of new extraction processes is the presence of other compounds in the final extract that might interfere with the downstream process. In this case, the YCH was determined (Figure 4D and Figure 5D) and it was shown that in the conditions used for the confirmation runs, the ratio between PAC and CH content was 1.47 and 1.33 for ChCl:LacA and ChCl:Glyc, respectively.

Due to the higher Y_PAC_, mDP and YPAC/YCH ratio that was possible to obtain with ChCl:LacA, this was chosen as the best candidate for the optimization of the extraction conditions. Nevertheless, it is noteworthy that if PAC depolymerization is completely undesired, the use of ChCl:Glyc might be beneficial in spite of the substantially lower Y_PAC_.

### 2.5. Extraction Conditions Optimization

**Figure 6:**
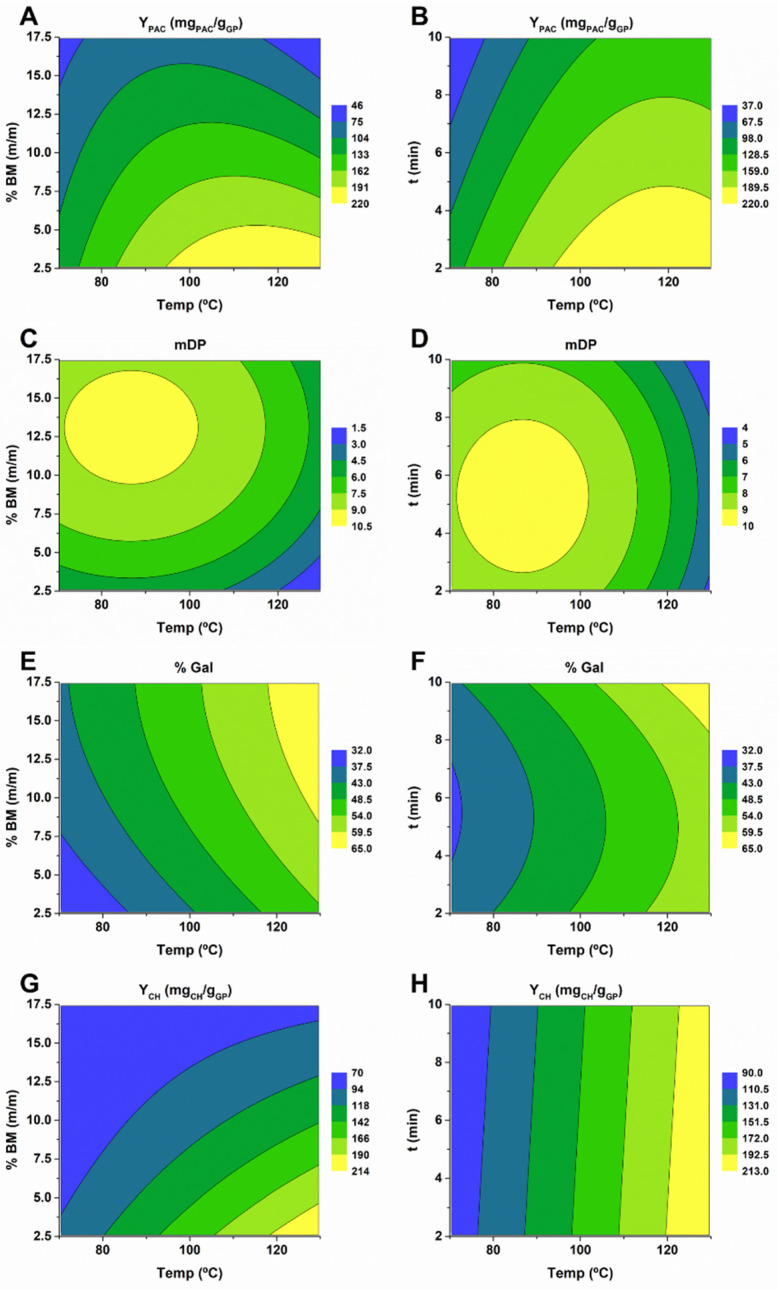
Contour plots obtained for the extraction conditions for the optimization of ChCl:LacA. (**A**) Y_PAC_ for t = 2 min; (**B**) Y_PAC_ for %BM = 2.5%; (**C**) mDP for t = 5.23 min; (**D**) mDP for %BM = 13%; € %Gal for t = 10 min; (**F**) %Gal for %BM = 17.5%; (**G**) Y_HC_ for t = 2 min; (**H**) Y_HC_ for %BM = 2.5 min.

MAE provides, as the main advantage, the considerable reduction in extraction time [28] that leads to considerable savings in energy consumption. When using MAE, there are three main parameters that can be optimized, namely, temperature (T), extraction time (t), and biomass percentage (%BM). To determine the effect of each parameter on the variables previously mentioned, while using ChCl:LacA, RSM was employed with 15 experimental points in which the parameters were varied from 70 to 130 °C for temperature, 2 to 10 min for extraction time, and 2.5 to 17.5 for %BM, as specified in Table S4 in the Supplementary Materials. For each experimental condition, Y_PAC_, mDP, %Gal, and Y_CH_ were determined. The corresponding polynomials are depicted in Equations (9), (10), (11), and (12), and the respective contour plots are shown in Figure 6.

Y_PAC_ = −372.0 + 10.41T + 3.717%BM − 12.48t − 0.1322T%BM + 1.033%BMt − 0.04235T^2^ (9)

mDP = −14.42 + 0.3721T + 0.955%BM + 0.742t − 0.00215T^2^ − 0.03668%BM^2^ − 0.0709t^2^ (10)

%Gal = 9.89 + 0.3010T + 1.511%BM − 3.439t + 0.00597Tt − 0.04068%BM^2^ + 0.276t^2^ (11)

Y_CH_ = −39.55 + 2.177T + 2.525%BM − 1.129t − 0.1126%BM + 0.1590%BMt + 0.1717%BM^2^ (12)

ANOVA analysis revealed that the resulting models for Y_PAC_, mDP, %Gal, and Y_CH_ all had *p*-values < 0.003, adjusted r^2^ values of 0.86, 0.86, 0.84, and 0.90, respectively, and predicted r^2^ values of 0.64, 0,68, 0.42, and 0.62, respectively. This indicates that despite the high adjusted r^2^, it is possible to obtain that the predicted r^2^ were lower than what should be expected, especially for %Gal, which might be indicative of some statistical fragility of the resulting quadratic models.

In Figure 6, the impact that these parameters have on Y_PAC_, mDP, %Gal, and YCH can be observed. In terms of Y_PAC_, the maximum value was obtained at 120 °C with 2.5% BM (Figure 6A). Interestingly, by increasing the % BM, the temperature at which maximum Y_PAC_ is obtained decreases. In addition, for any given temperature, the increase in extraction time leads to a decrease in Y_PAC_ (Figure 6B), demonstrating that thermal degradation is present, regardless of the temperature employed, further sustaining the necessity of carefully controlling temperature and extraction time in order to preserve Y_PAC_.

As far as mDP is concerned, the maximum values were obtained with a temperature of 86 °C and a % BM of 13%, clearly showing the negative effect that higher temperatures have on mDP (Figure 6C). In fact, using the conditions at which the maximum Y_PAC_ is obtained, the resulting mDP was 2.4, which is proof of almost complete PAC depolymerization at higher temperatures and under high acid content. Furthermore, extraction time has a positive effect on mDP up until 5.23 min, after which a decrease is verified (Figure 6D).

The highest values for %Gal were obtained with the main values tested for all parameters, which overlapped with low values of Y_PAC_ and mDP that might be indicative of a higher resistance of galloylated monomers to thermal degradation (Figure 6E,F). The results obtained for Y_CH_ were in line with those expected, increasing considerably with temperature and decreasing with higher % BM (Figure 6G). Extraction time had no effect on the overall Y_CH_ (Figure 6H).

Considering that the most important characteristics are Y_PAC_ and mDP, the best results were obtained while using 99.2 °C, 8.3% BM, and 3.56 min, which should result in Y_PAC_ of 152 mg_PAC_/g_GP_ and a mDP of 8.13. The confirmation runs resulted in a Y_PAC_ of 135 mg_PAC_/g_GP_ and a mDP of 7.19, which is in reasonable agreement with the expected values, validating the models used.

This was a slight improvement on the extraction yield obtained in our previous work, from 126 mg_PAC_/g_GP_ in which extraction was made through conventional maceration using the same raw material [14]. Despite the inexistence of considerable improvements in extraction yield, it is noteworthy that the extraction time was drastically reduced from 1 h to 3.56 min.

Furthermore, the use of MAE has been described as one of the best candidates for the reduction in environmental impact of extraction processes while improving overall extraction yield even after scale-up to pilot scale extraction [29].

Considering the importance of solvent stability throughout the MAE process, and taking into account the possibility of reusing it is of utmost importance as far as sustainability is concerned, the effect of using the optimized extraction conditions twice on the solvent was assessed by ^13^C NMR spectroscopy comparing the ES spectra with literature data [30].

**Figure 7:**
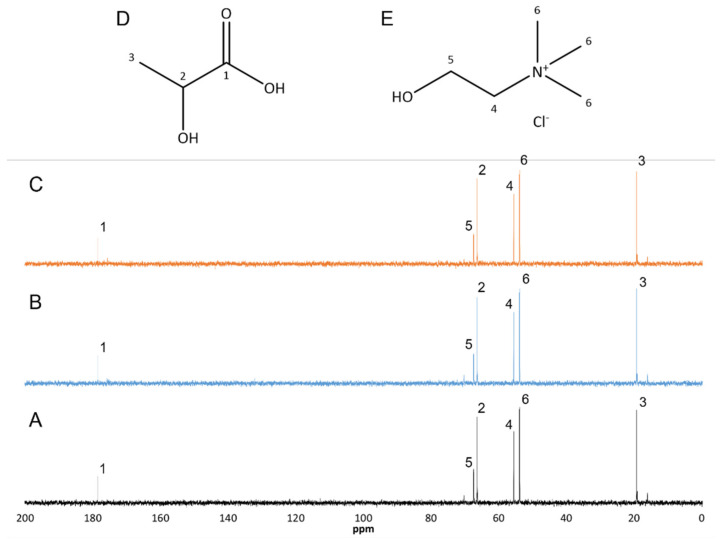
^13^C NMR spectra of ChCl:LacA:H_2_O (36:39:25) (m:m:m). (**A**) Control; (**B**) Incubated once; (**C**) Incubated twice; (**D**) Lactic acid molecular structure; (**E**) Choline chloride molecular structure. 1–6–^13^C NMR peak attribution.

As can be observed in Figure 7, no differences were observed in the corresponding ^13^C NMR spectra even after the solvent had been submitted twice (Figure 7C) to an incubation at 99.2 °C for 3.56 min on a microwave extractor.

## 3. Materials and Methods

### 3.1. Eutectic Solvent Preparation

ESs were prepared by mixing 37.5% (*m*/*m*) of HBA and HBD with 25% (*m*/*m*) of water and agitating at 40 °C until a continuous phase was obtained. Tested HBAs were choline chloride (ChCl), betaine (Bet), and proline (Pro), and tested HBDs were urea (Ur), malic acid (MalA), citric acid (CitA), lactic acid (LacA), glucose (Glu), and glycerol (Glyc).

### 3.2. Proanthocyanidin Reference Material

PAC reference material was extracted from white GP that was a result of mixing several grape varieties from the Douro region in Portugal during the 2019 harvest. The extraction method was described by Neto et al. [14] with slight modifications. Briefly, GP was frozen at −20 °C and freeze-dried followed by defatting with dichloromethane in a Soxhlet for 6 h. GP was then extracted three times with aqueous acetone 70% (*v*/*v*) at a solid:liquid proportion of 1:10 for 2 h, at room temperature. After filtration under vacuum, acetone was removed in a rotary evaporator (Buchi, Flawil, Switzerland) until a precipitate was formed, which was removed by centrifugation and discarded. The remaining soluble fraction (representing around 10% (*m*/*m*) of the defatted GP) was freeze-dried and used as reference material for the screening of PAC stability in ESs.

### 3.3. Proanthocyanidin Purification

High molecular weight PACs were purified and used for PAC quantification with acid butanol assay following the method described by Neto et al. [14]. Briefly, 2 g of the PACs reference material isolated above were dissolved in 20 mL of methanol and loaded into a glass column packed with 16 × 100 mm^2^ of Toyopearl HW-40 resin equilibrated with methanol. Sample was washed with 300 mL of methanol and 250 mL of methanol with 30% (*v*/*v*) of acetone to remove sugars and low molecular weight phenolic compounds and high molecular weight PACs were eluted with 150 mL of acetone with 30% (*v*/*v*) of water. Solvent was then removed in a rotary evaporator (Buchi, Flawil, Switzerland) until complete dryness, resuspended in distilled water, freeze-dried, and kept in a desiccator until needed.

### 3.4. Acid Butanol Assay

PAC quantification was made following the acid butanol assay described by Porter et al. [17]. Briefly, 82.5 µL of purified PAC/sample dissolved in methanol was mixed with 500 µL of butanol reagent (butanol with 5% (*v*/*v*) of concentrated hydrochloric acid) and 18 µL of ammonium iron(III) sulfate dodecahydrate solution (20 mg/mL prepared in aqueous hydrochloric acid (2 M)) in pressure and temperature resistant tubes that were then incubated at 100 °C for 50 min. The absorbance of the resulting solution was measured at 520 nm.

### 3.5. Determination of Mean Degree of Polymerization and Galloylation Percentage by Phloroglucinolysis

The PAC’s mDP and %Gal were determined following the phloroglucinolysis method described by Kennedy et al. [31] with slight modifications. Briefly, 10 to 100 mg of sample, depending on PAC concentration, were dissolved in 1.0 mL of a freshly prepared methanol solution containing 50 g/L of phloroglucinol, 10 g/L of ascorbic acid, and 0.1 M of hydrochloric acid. Insolubilized material was removed by centrifugation, after which 400 µL of the supernatant were transferred to pressure resistant vials and incubated at 50 °C for 1 h followed by the addition of 2 mL of 40 mM sodium acetate aqueous solution to stop the reaction. Depolymerization products were quantified by high performance liquid chromatography (HPLC) in a Accela 80 Hz (Thermo Fisher Scientific, San Jose, CA, USA) (Figure S1 in Supplementary Materials) and peak attribution was made by electrospray ionisation mass spectrometry (ESI-MS) in a LCQ Fleet ion trap mass spectrometer (ThermoFinnigan, San Jose, CA, USA) (Table S1 in Supplementary Materials) operated as described elsewhere [32]. mDP was calculated by dividing the sum of terminal and extension units by the sum of terminal units and the %Gal was calculated by dividing the sum of galloylated units by the sum of all units.

### 3.6. Screening of Proanthocyanidin Stability in Eutectic Solvents

The thermal stability of PACs in a MAE process was evaluated by dissolving 80 mg of PAC reference material in 4 g of selected ESs and agitated under magnetic stirring at room temperature until complete dissolution. The prepared solutions were then divided into four aliquots of 1 g with one serving as the control and the remaining ones incubated for 10 min at 70, 100, and 130 °C in a microwave synthesis reactor (Monowave 300 from Anton Paar (Madrid, Spain)). PAC content was calculated by the acid butanol assay.

### 3.7. Screening of Proanthocyanidin Extractability with Eutectic Solvents

PAC extractability with ESs in a MAE process was evaluated by mixing 300 mg of GP with 2.7 g of the different ESs and incubating it at 100 °C and 600 rpm in a microwave extractor, a Monowave 300 from Anton Paar (Graz, Austria) for 10 min. PAC content was calculated by the acid butanol assay.

### 3.8. Screening of Eutectic Solvents Stability

The thermal stability of ESs in a MAE process was evaluated by incubating 1 g of each ES in a microwave extractor, a Monowave 300 from Anton Paar (Graz, Austria) for 10 min at 130 °C.

### 3.9. Eutectic Solvent Composition Optimization

ES solvent composition was optimized with response surface methodology (RSM) using the mixtures function with D-optimal design in Expert Design v12 from StateEase (Minneapolis, MN, USA). The experiment consisted of 16 experimental points in which the mass fractions were varied as follows: ChCl:LacA-0 to 0.7 for ChCl, 0 to 0.9 for LacA and 0 to 0.7 for water; ChCl:Glyc-0 to 0.7 for ChCl, 0 to 1 for Glyc, and 0 to 0.5 for water. Experimental solvent compositions and experimental results are detailed in Tables S2 and S3 in the Supplementary Materials. Extractions were performed under continuous agitation at 600 rpm with 10% (*m*/*m*) of GP at 100 °C and confirmation runs were performed in triplicate for the solvent compositions that resulted in the best Y_PAC_ and mDP. PAC content was calculated by the acid butanol assay and PAC mDP was determined by phloroglucinolysis.

### 3.10. Extraction Conditions Optimization

Extraction conditions were optimized with RSM following Box–Behnken Design in Expert Design v12 from StateEase (Minneapolis, MN, USA). The experiment consisted of 15 experimental points in which T, %BM, and t assayed values ranged from 70 to 130 °C, 2.5 to 17.5% (*m*/*m*), and 2 to 10 min, respectively. Experimental extraction conditions and experimental results are detailed in Table S4 in the Supplementary Materials. Extractions were performed under continuous agitation at 600 rpm and the solvent composition was the one from which the highest YPAC and mDP values were obtained and confirmation runs were performed in triplicate for the experimental conditions that resulted in the best YPAC and mDP. PAC content was calculated by the acid butanol assay and PAC mDP was determined by phloroglucinolysis.

### 3.11. NMR Analysis

Solvent stability was evaluated by ^13^C NMR with spectra being obtained with a Bruker Avance 300 (Wissembourg, France) at 75.47 MHz using trimethylsilyl propanoic acid (TMSP) as the internal reference and deuterated water as the solvent.

## 4. Conclusions

Direct comparisons with published work is always problematic since each type of biomass has its own characteristics that have a direct impact on the characteristics of the final extract. Nevertheless, in our previous work [14] that used the same GP as the raw material, it was already shown that the improvement could be achieved by replacing conventional solvents with ESs, namely, mixtures of ChCl:Glyc with ethanol. In the present work, it was shown that by using an ES composed of x_ChCl_ = 0.36, x_LacA_ = 0.39, and x_water_ = 0.25 in combination with MAE at 99.2 °C, with 8.3% BM, it was possible to considerably reduce the extraction time from 1 h to 3.56 min while obtaining slightly higher Y_PAC_ (135 vs. 126 mg_PAC_/g_GP_) and mDP (7.2 vs. 6.5).

Herein, the possibility of combining ESs with MAE in the extraction of PACs from GP was achieved, being demonstrated that the combination of choline chloride with lactic acid was the most effective for the PAC extraction yield and, despite the occurrence of some depolymerization, also enabled us to achieve the highest mDP. Despite the initial necessary investment, this should be offset by the reduction in the energy necessary for the extraction process and the higher extraction yield.

The importance of PACs for industrial applications has increased considerably in recent years due to their ability to replace harmful chemicals, and therefore, its demand is also expected to increase. Herein, it was shown that better and more efficient methods can be developed by combining ESs with MAE, more specifically, ChCl:LacA-based ESs. 

## Data Availability

Not applicable.

## Supplementary Materials

The following are available online, Figure S1: Representative chromatogram of phloroglucinolysis products for proanthocyanidin mean degree of polymerization determination, Table S1: Compounds identified from phloroglucinolysis products and the respective retention times and molecular ion masses, Table S2: Solvent composition and experimental results of solvent composition optimization of ChCl–LacA–water, Table S3: Solvent composition and experimental results of solvent composition optimization of ChCl–Glyc–water, Table S4: Extraction conditions and experimental results of extraction conditions optimization of ChCl–LacA–water.

## Sample Availability

Not available.
