# Supplementary material for: Impact of Eutectic Solvents Utilization in the Microwave Assisted Extraction of Proanthocyanidins from Grape Pomace
Source: Molecules. 2021 Dec 31;27(1):246. doi: 10.3390/molecules27010246 (PMC8746617; doi:10.3390/molecules27010246)
Supplement: Supplementary file 1 [file molecules-27-00246-s001.zip › molecules-1516924-supplementary.pdf]

Impact of Eutectic Solvents Utilization in the Microwave Assisted Extraction of Proanthocyanidins from grape pomace

Rodrigo T. Neto [1], Sónia A. O. Santos [1], Joana Oliveira [2] and Armando A. J. Silvestre [1,*]

[1] CICECO - Aveiro Institute of Materials, Chemistry Department University of Aveiro, Campus de Santiago, 3810-193 Aveiro, Portugal

[2] REQUIMTE - Laboratório Associado para a Química Verde, Department of Chemistry and Biochemistry, Faculty of Sciences, University of Porto, Rua do Campo Alegre, 687, 4169-007 Porto, Portugal

* Correspondence: armsil@ua.pt; Tel.: + 351 234 370 711; Fax: + 351 234 370 084

Figure S1 – Representative chromatogram of phloroglucinolysis products for proanthocyanidin mean degree of polymerization determination. 1 – phloroglucinol adduct of catechin/epicatechin; 2 – phloroglucinol adduct of catechin/epicatechin; 3 – catechin; 4 – phloroglucinol adduct of catechin gallate/epicatechin gallate; 5 – epicatechin; 6 – catechin gallate/epicatechin gallate

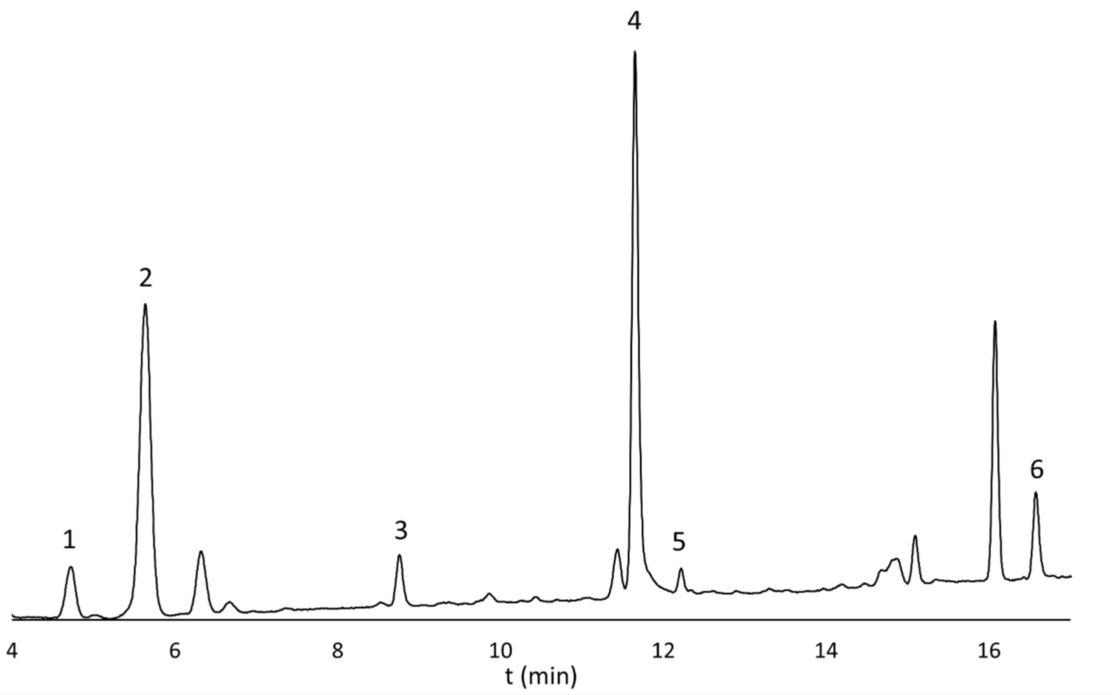

Table S1 – Compounds identified from phloroglucinolysis products and the respective retention times and molecular ions masses.

No	Compound	t_R (min)	$[M-H]^-$ (m/z)
1	phloroglucinol adduct of catechin/epicatechin	4.72	413.19
2	phloroglucinol adduct of catechin/epicatechin	5.64	413.19
3	Catechin	8.76	289.08
4	phloroglucinol adduct of catechin gallate/epicatechin gallate	11.65	565.20
5	epicatechin	12.22	289.08
6	catechin gallate/epicatechin gallate	16.58	441.09

Table S2 – Solvent composition and experimental results of solvent composition optimization of ChCl-LacA-water.

				Y_{PAC} (mg_{PAC}/g_{GP})		mDP		%Gal		Y_{CH} (mg_{CH}/g_{GP})	
Run	x_{ChCl}	x_{LacA}	x_{H2O}	Measured	Predicted	Measured	Predicted	Measured	Predicted	Measured	Predicted
1	0.490	0.408	0.102	101.7	113.7	9.94	10.60	38.11	38.32	60.9	66.2
2	0.248	0.602	0.150	113.0	115.1	9.52	10.23	38.03	38.19	68.8	63.6
3	0.492	0.201	0.307	114.7	116.4	10.79	10.39	34.86	35.54	82.0	79.1
4	0.700	0.240	0.060	72.8	77.2	9.18	8.70	33.58	35.73	52.0	55.8
5	0.698	0.000	0.302	70.8	71.0	7.57	7.75	31.00	31.52	62.5	60.0
6	0.505	0.000	0.495	73.2	80.5	7.47	7.85	31.37	31.49	65.3	73.3
7	0.502	0.000	0.498	74.5	80.3	7.40	7.83	31.24	31.47	66.8	73.5
8	0.700	0.237	0.063	89.4	77.5	8.82	8.70	38.04	35.69	56.8	56.0

9	0.000	0.405	0.595	58.3	63.4	6.03	6.25	30.72	31.27	95.0	89.5
10	0.000	0.399	0.601	60.6	62.1	5.85	6.17	31.47	31.17	87.8	89.7
11	0.000	0.800	0.200	75.2	71.5	7.45	7.02	34.28	34.59	54.3	45.01
12	0.000	0.599	0.401	78.4	86.3	7.24	7.79	33.34	33.58	63.3	76.2
13	0.306	0.381	0.313	132.2	127.7	11.03	10.87	37.41	36.83	83.1	83.9
14	0.700	0.000	0.300	73.7	70.7	7.74	7.74	32.27	31.51	67.2	59.8
15	0.000	0.800	0.200	75.1	71.4	7.39	7.02	35.10	34.59	39.9	45.01
16	0.247	0.216	0.537	119.2	97.6	10.15	8.66	34.04	33.37	101.0	90.2

Table S3 – Solvent composition and experimental results of solvent composition optimization of ChCl-Glyc-water.

				Y_{PAC} (mg_{PAC}/g_{GP})		mDP		%Gal		Y_{CH} (mg_{CH}/g_{GP})	
Run	x_{ChCl}	x_{Glyc}	x_{H2O}	Measured	Predicted	Measured	Predicted	Measured	Predicted	Measured	Predicted
1	0.000	1.000	0.000	63.3	62	7.62	7.65	39.80	39.48	69.9	63.2
2	0.321	0.479	0.200	74.2	71.1	7.45	7.37	34.90	34.28	70.8	66.0
3	0.000	0.758	0.242	70.0	72.6	7.60	7.67	35.58	36.18	81.1	79.1
4	0.501	0.000	0.499	71.7	76.0	7.13	7.14	31.21	31.97	71.2	66.2
5	0.699	0.000	0.301	66.2	70.7	7.85	7.96	31.00	32.21	69.1	69.5
6	0.242	0.259	0.499	68.2	70.8	6.63	6.66	32.49	31.93	61.5	67.3
7	0.488	0.229	0.283	74.5	73.3	7.47	7.50	33.26	32.92	65.8	67.3
8	0.659	0.281	0.060	47.87	46.94	7.06	7.18	32.33	32.10	54.5	51.1
9	0.502	0.498	0.000	47.11	45.78	6.73	6.79	31.85	33.55	44.03	44.57
10	0.000	0.502	0.498	60.7	62.5	6.57	6.59	31.89	32.24	70.6	74.1
11	0.000	1.000	0.000	63.3	62.0	7.71	7.65	40.00	39.48	58.1	63.2
12	0.000	0.502	0.498	66.1	62.5	6.65	6.59	32.28	32.24	77.2	74.1
13	0.657	0.283	0.060	48.02	47.04	7.33	7.17	33.88	32.11	53.6	51.1
14	0.500	0.000	0.500	81.4	76.0	7.15	7.14	32.83	31.97	69.1	66.1
15	0.246	0.754	0.000	51.5	55.8	7.04	7.03	35.77	36.40	44.86	51.2
16	0.699	0.000	0.301	73.6	70.7	7.80	7.96	32.18	32.21	62.0	69.5

Table S4 – Extraction conditions and experimental results of extraction conditions optimization of ChCl-LacA-water.

				Y_{PAC} (mg_{PAC}/g_{GP})		mDP		%Gal		Y_{CH} (mg_{CH}/g_{GP})	
Run	temp (ºC)	%BM	t (min)	Measured	Predicted	Measured	Predicted	Measured	Predicted	Measured	Predicted
1	130	9,84	10	112.8	109.8	3.293	3.415	63.8	61.0	144.1	145.3
2	100	17,51	10	127.8	135.1	7.08	6.74	50.7	53.2	103.3	94.4
3	100	2,621	10	121.7	122.8	2.959	3.524	44.89	42.88	135.3	149.4
4	100	9,99	6	135.8	137.5	8.22	8.72	44.27	43.93	113.0	110.8
5	100	2,471	2	201.4	202.1	3.949	4.280	41.24	38.90	142.6	156.2
6	100	10,22	6	139.3	136.8	9.09	8.77	43.43	44.09	111.4	109.8
7	70	10,63	2	77.4	87.3	6.83	7.97	34.91	37.50	76.3	76.4
8	130	17,72	6	53.9	61.7	2.996	4.543	58.1	57.0	84.9	92.9
9	130	10,30	2	141.3	123.1	5.24	4.386	58.6	56.6	137.4	138.0
10	100	10,30	6	150.2	136.5	8.89	8.79	44.06	44.14	105.6	109.5
11	100	17,47	2	84.3	90.5	8.24	7.63	47.10	49.41	90.4	81.3

12	70	10,01	10	79.8	72.3	7.32	6.98	37.09	39.43	82.3	81.1
13	70	17,46	6	90.3	85.8	8.85	8.15	41.68	36.77	72.0	81.6
14	70	2,569	6	73.8	76.0	4.983	4.878	26.84	26.40	109.2	95.9
15	130	2,626	6	158.0	171.5	2.193	1.398	41.73	46.68	223.3	208.6
